# PARTNERED EVALUATION OF LONGITUDINAL IMPACT OF STAR-VA: IMPLICATIONS FOR PROGRAM SUSTAINABILITY

**DOI:** 10.1093/geroni/igz038.2351

**Published:** 2019-11-08

**Authors:** Michele J Karel, Lisa A Minor, jenefer M Jedele, Orna Intrator, Kim J Curyto

**Affiliations:** 1 Office of Mental Health and Suicide Prevention, Veterans Health Administration, Washington, District of Columbia, United States; 2 Geriatrics and Extended Care, Department of Veterans Affairs, Washington, District of Columbia, United States; 3 Serious mental Illness Treatment Resource and Evaluation Center, Office of Mental Health and Suicide Prevention, Department of Veterans Affairs, Ann Arbor, Michigan, United States; 4 GECDAC at Canandaigua VA Medical Center and Department of Public Health Sciences, University of Rochester, Rochester, New York, United States; 5 Center for Integrated Healthcare, VA Western NY Healthcare System, Batavia, New York, United States

## Abstract

Between 2013 and 2018, 86 Community Living Center (CLC) teams received STAR-VA training and consultation and implemented the intervention. Program evaluation has consistently demonstrated that STAR-VA Veteran training cases experience significantly decreased frequency and severity of target behaviors, symptoms of depression, anxiety, and agitation. VA Central Office leaders responsible for ongoing STAR-VA implementation and evaluation sought to understand the program’s systemic impact on important outcomes, if outcomes are maintained, and factors influencing variation in sustained adherence to STAR-VA. This presentation will describe the rationale for and methods used to develop a valid quality improvement metric to assess behavioral symptoms of dementia, to compare Veteran- and site-level outcomes at trained and untrained CLCs, and to identify factors associated with sustained STAR-VA implementation. This partnered evaluation, combining quantitative and qualitative methodologies, is critical for informing operational strategies for promoting STAR-VA and supporting its sustained implementation in VA CLCs.

